# Overcoming immunotherapy barriers in pediatric brain tumors: epigenetic strategies

**DOI:** 10.3389/fonc.2026.1857121

**Published:** 2026-07-24

**Authors:** Ashley R. Tetens, Jordyn Craig-Schwartz, Tyler R. Findlay, Michael A. Koldobskiy

**Affiliations:** 1 Center for Epigenetics, Johns Hopkins University School of Medicine, Baltimore, MD, United States; 2 Pediatric Oncology, Sidney Kimmel Comprehensive Cancer Center, Johns Hopkins University School of Medicine, Baltimore, MD, United States; 3 Department of Physiology, Pharmacology, and Therapeutics, Johns Hopkins University School of Medicine, Baltimore, MD, United States

**Keywords:** brain tumors, epigenetics, immunotherapy, pediatric oncology, tumor immune microenvironment

## Abstract

The advent of cancer immunotherapy has led to dramatically improved outcomes in several immunogenic adult cancers. Similar successes have been seen in some pediatric cancers, but only in specific settings. A particular challenge has been the application of immunotherapy approaches to pediatric brain tumors, with early clinical experience showing promise but few durable responses. Key barriers to immunotherapy arise from distinctive features of pediatric brain tumors, including low tumor immunogenicity, an immunosuppressive tumor microenvironment, and impaired effector cell function. Additionally, the application of immunotherapies to the confined space of the CNS must be carefully calibrated to avoid neurological toxicity. Unlike immunogenic adult cancers, many pediatric brain tumors have a paucity of genetic mutations and are driven by epigenetic alterations. Tumor subtypes with the same genetic driver can have markedly different tumor immune microenvironments, illustrating that non-genetic factors contribute to immune dysregulation. Here, we review key features of the tumor immune microenvironments of malignant pediatric brain tumors and consider how these features serve as barriers to successful application of immunotherapy. We evaluate evidence that pharmacologic manipulation of the epigenome can be leveraged to shape the tumor immune microenvironment and consider combinatorial approaches using epigenetic-targeted therapies and immunotherapies to inform future clinical studies of pediatric brain tumors.

## Introduction

Immunotherapy has revolutionized the treatment of several adult malignancies, leading to durable responses in highly aggressive cancers such as melanoma, non-small cell lung cancer, and renal cell carcinoma (1–4). In pediatric oncology, immunotherapy has also produced therapeutic advances in select settings, such as chimeric antigen receptor (CAR) T-cell therapy for B-cell acute lymphoblastic leukemia (5). However, successful application of immunotherapy to the treatment of pediatric solid tumors, and particularly pediatric brain tumors, has remained elusive.

Immunotherapies in pediatric patients with brain tumors pose the greatest challenge, as these therapies must elicit an antitumor immune response while avoiding excessive neuro-inflammation and preserving neurological function, and they must overcome anatomic and physiologic constraints such as the blood-brain barrier (6). Further, the tumor microenvironments (TME) in many pediatric brain tumors are also poised to oppose both the natural immune system and engineered immunotherapies. While each cancer type has distinct TME properties, common immunosuppressive features across pediatric brain tumors include low neoantigen burden leading to reduced immunogenicity, downregulation of major histocompatibility complex (MHC) molecules leading to impaired antigen presentation, and recruitment or polarization of myeloid cells that support tumor growth (7). Thus, immunotherapies aimed at enhancing effector cell function must overcome an environment that is configured to limit their activity.

**Figure 1:**
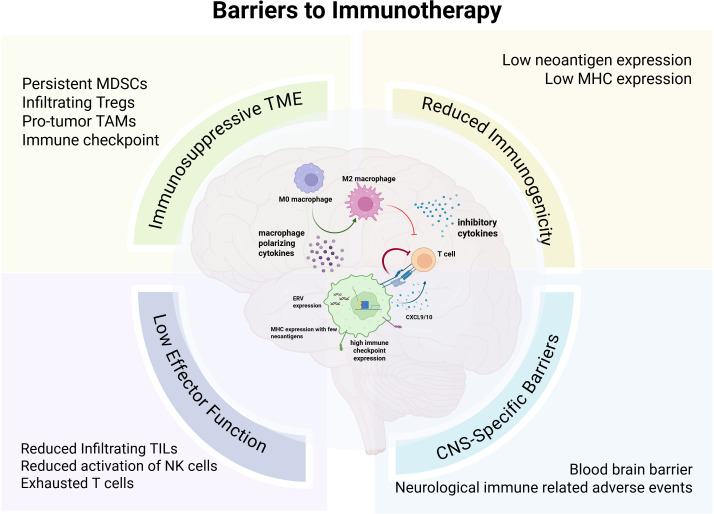
Barriers to immunotherapy include an immunosuppressive tumor microenvironment (TME) (i.e. myeloid derived suppressor cells [MDSCs], infiltrating regulatory T cells [Tregs], tumor-associated macrophages [TAMs], and immune checkpoint expression), reduced immunogenicity (low neoantigen expression, low MHC expression), low effector function (i.e. reduced presence of tumor infiltrating lymphocytes [TILs], reduced activation of natural killer [NK] cells, and exhausted T cells), and CNS-specific barriers (i.e. blood brain barrier, and neurological immune related adverse events).

Clinical trials of immunotherapies for pediatric brain tumors have encompassed multiple modalities, including immune checkpoint blockade, CAR-T cells, dendritic cell vaccines, and oncolytic viruses. Across these distinct immunotherapy modalities, common barriers to clinical benefit have emerged. Recurrent barriers, several of which are appreciated as universal barriers to immunotherapy, include the immunosuppressive TME, reduced immunogenicity of the cancer cells, impaired effector cell function, and CNS-specific barriers, such as the blood-brain barrier and the emergence of neurological immune-related adverse events (N-irAEs) (**Figure 1**) (8).

Epigenetic therapies offer a promising strategy to address these barriers. Preclinical and clinical evidence suggests that drugs that target DNA methylation, histone acetylation, or histone methylation can increase tumor immunogenicity and antigen presentation, induce viral mimicry programs, and reprogram the TME (9–14). These effects suggest a promising complementary role for epigenetic therapy in overcoming immunotherapy barriers imposed by pediatric brain tumors.

In this review, we discuss the clinical experience with immunotherapies in pediatric brain tumors and evaluate ways in which epigenetic therapies can be leveraged to overcome key biological and clinical barriers. We will discuss both FDA-approved and emerging small molecule agents that target the epigenome and highlight rational combinations of epigenetic and immune therapies that could inform the development of future trials.

## Tumor immune microenvironment of pediatric brain cancers

Many of the barriers that emerge during immunotherapy use in brain tumors are extensions of the innate features of the TMEs in these cancers. Understanding the mechanisms by which a cancer evades the endogenous immune system can provide insight into its capacity for evasion of immunotherapy. Over the past decade, there have been increased efforts to characterize the tumor microenvironments of several pediatric brain cancers (15). This has been an essential undertaking, given that there may be differences between the mature adult CNS immune system and the immature pediatric immune system that could underlie differential responses to immunotherapy. Further, there is heterogeneity in the TME both between cancer types and within a given cancer type. This is true even in cancers with the same driver mutation, such as between subgroups of SMARCB1-deficient atypical teratoid rhabdoid tumors (ATRT) (16).

**Figure 2:**
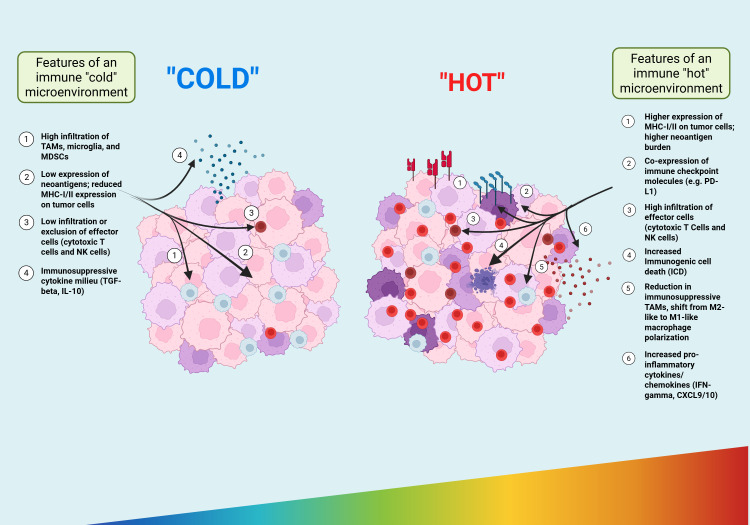
Tumor immune microenvironments lie on a continuum from immune “cold” or excluded to immune “hot” or altered. Features of immune “cold” tumors include 1) high infiltration of immunosuppressive myeloid cells, 2) low expression of neoantigens and reduced MHC Class I/II presentation, 3) low infiltration of effector cells (cytotoxic T cells and natural killer (NK) cells), and 4) an immunosuppressive cytokine milieu (TGF-β and IL-10). In contrast, immune “hot” tumors have 1) a higher neoantigen burden and comparably higher expression of MHC-I/II on tumor cells, 2) co-expression of immune checkpoint molecules (i.e. PD-L1), 3) increased infiltration of effector cells, 4) increased immunogenic cell death, 5) reduction in immunosuppressive myeloid cell populations, and 6) increased pro-inflammatory cytokines and chemokines (i.e. IFN-gamma, CXCL9/10).

This suggests that non-genetic factors contribute to the establishment and maintenance of distinct immune landscapes. Indeed, it is understood that several features of the immune microenvironment, including the innate immunogenicity of the tumor and the composition and activity of immune cells in the TME, are influenced by non-genetic factors, particularly epigenetic factors, and thus may be amenable to pharmacologic modulation (17). Below we relate the tumor microenvironments of different pediatric brain tumors to highlight relevant barriers that might emerge with immunotherapy use. We include a consideration of the microenvironments of different subgroups of the same diagnosis to highlight how epigenetic features can shape the microenvironment to be more ‘hot’ or ‘cold’ (**Figure 2**) (18). **Table 1** provides a summary of key TME features of selected malignant pediatric brain tumors that illustrate these recurrent barriers. Below we highlight specific cases of ATRT, ependymoma, and oncohistone-mutant diffuse gliomas as examples of subtype-dependent differences in immune environment.

**Table 1:** Description of immune phenotypes and status of immune infiltration of several pediatric brain tumors.

Tumor type/subgroup	Immune phenotype	Immune infiltration/signatures	Key mechanisms	Key references
High-grade glioma				
Diffuse pediatric-type pHGG, H3/IDH-wild type	Altered (active yet immunosuppressed)	• Heterogeneous group, variable immune infiltration • Relatively higher CD8+ T-cell infiltration compared to H3-mutant • Dominated by immunosuppressive programs limiting effector function • Higher mutation burden in some subsets, particularly mismatch-repair deficient	• Elevated CD163+ M2-polarized macrophages • CD8+ T cell infiltration present but functionally impaired • increased PD-L1	(15, 43, 46–48)
IDH-mutant glioma	Cold/immunosuppressed	• Reduced CD8+ T-cell infiltration • Features consistent with T-cell exclusion • Microglia-dominant myeloid compartment	• D-2HG can impair T-cell activation via STAT1 suppression, reduced CXCL9/10, direct inhibition of T cell proliferation	(49–53)
H3 K27M (Diffuse midline glioma)	Cold/desert	• Minimal T-cell infiltration • Extremely low tumor mutational burden • Dominated by immunoquiescent myeloid populations	• Immunoquiescent myeloid cells; limited cytokine production for T cell trafficking; low surface MHC-I leading to poor antigen presentation	(42, 43, 54)
H3 G34R	Cold/altered	• Sparse immune infiltration, limited lymphocyte populations • Heterogeneous myeloid compartment	• Sparse lymphocytic infiltration • Epigenetic dysregulation of immune-related genes modulate local environment	(42, 44, 45)
Embryonal tumors				
Medulloblastoma (MB): SHH	Altered - myeloid dominant	• Highest TAM infiltration and expression of inflammation-related genes among MB subgroups • Low T cell infiltration	• Increased TAMs • Expression of CD163, CSF1R axis, pro-tumor polarization	(55–58)
MB: WNT	Cold	• Low T-cell infiltration despite increasingly porous blood-brain barrier in this subtype • Higher PD-L1 expression than other subgroups	• WNT signaling can mediate CD8+ T cell exclusion, Treg promotion, and PD-L1 expression	(59–62)
MB: group 3 and 4	Cold	• Low immune infiltration, but group 3 has higher proportion of CD8+ • Suppressive immune stromal environment	• CD47 mediated escape from phagocytosis • Immunosuppressive cytokine and checkpoint pathways: PD-L1, CTLA4, TGF-β.	(59, 63)
ATRT-MYC	Hot (inflamed with adaptive checkpoints)	• Increased CTL infiltration and high cytolytic score comparable to immunogenic adult tumors • concomitant checkpoint upregulation	• High CD8+ CTLs; granzyme/perforin program • increased checkpoint expression • ERV expression associated with cytolytic activity	(25, 26)
ATRT-SHH	Cold	• Low CD8+ in IHC but detected by methylCIBERSORT • Limited cytolytic activity • TAMs abundant	• Highest level of PD-L1-negative CD68+ myeloid cells • Lower immune gene expression vs. ATRT-MYC • Shh signaling may drive pro-tumoral myeloid polarization	(25, 26, 64)
ATRT-TYR	Mixed	• Low CD8+ cell infiltration • Shared immune response gene sets with ATRT-MYC	• Treg, NK cell, and B cell infiltration	(25, 26, 64)
Ependymoma				
Ependymoma, posterior fossa group A (PFA-EPN)	Cold (myeloid-dominant; immunosuppressive)	• Low mutation burden/neoantigens, • Low CD8+ infiltration • TAMs with M2-like immunosuppressive phenotype	• EZHIP overexpression; global H3K27me3 loss; immunosuppressive chromatin landscape • Myeloid infiltration with IL-6/STAT3 axis and hypoxia-myeloid niches spatial hypoxia myeloid cells in perivascular/necrotic regions	(29, 30, 32–35)
Ependymoma, supratentorial, ZFTA fusion-positive (ST-EPN-ZFTA)	Altered (immune-active but exhausted/suppressed)	• Higher CD8+ density vs posterior fossa by IHC • infiltrating T cells show PD-1 and functional exhaustion; PD-L1 on tumor and myeloid cells	• Constitutive NF-κB activation via fusion oncoprotein; pro-inflammatory but immunosuppressive TME • MDSC recruitment	(33, 36–38)
Ependymoma, supratentorial, YAP1 fusion-positive (ST-EPN-YAP1)	Altered (cold)	• Lower PD-L1 than ST-EPN-ZFTA • Low overall immune cell infiltration	• Less immune-active phenotype than ST-EPN-ZFTA in reported cohort	(38)

### Subgroups of atypical teratoid rhabdoid tumor

ATRT is an embryonal tumor of childhood, defined by bi-allelic inactivation of the ATP-dependent chromatin remodeling complex, SMARCB1 (19, 20). ATRT is divided into three subgroups based on clinical and molecular features. Despite a consistent driver mutation, there are subgroup-specific differences in DNA methylation, enhancer networks, and transcription (21–23). Downstream of these epigenetic perturbations lie dissimilarities in the TME, emphasizing the impact of location, age, and modifiable non-genetic factors on the microenvironment.

Compared to other pediatric brain tumors, ATRT is considered to have a TME that is defined by a greater infiltration of effector cells, thus making it more immunologically active (16, 24). The different subgroups of ATRT exist on a continuum of immune infiltration and activity, however. Generally, rhabdoid tumors have a dynamic and varied immune microenvironment, consisting of M2-like macrophages, NK cells, and T cells (25). While tumor associated macrophages (TAMs) make up the highest proportion of the immune milieu, the TYR and MYC subgroups also have a dominating CD8+ T cell population that the SHH subgroup lacks (25, 26). Expectedly, the immune infiltrate in ATRT-SHH samples fails to exert a cytolytic effect, with low expression of granzyme and perforin genes, while ATRT-MYC and –TYR have a higher cytolytic score, on par with known immunogenic adult tumors (25, 27). Higher T cell infiltration and cytolytic activity is countered by a parallel increase in immune checkpoint molecules, suggesting that the application of immune checkpoint blockade therapy may have utility in subgroups with higher baseline effector infiltration (25, 26).

ATRT samples with high cytolytic activity also had a high expression of sense and anti-sense endogenous retroviral (ERV) expression, suggesting that ERV activity may aid T cell recruitment and cytolytic activity (25). Indeed, ERV expression has been linked to activation of interferon signaling in other cancers (9). Although ERV expression is dependent on SMARCB1 inactivation, the observation that ATRT-SHH exhibits low ERV expression and low cytolytic suggests SMARCB1 inactivation is necessary but not sufficient to induce ERV expression (25). It is well established that ERV expression can be induced by epigenetic modulation, raising the possibility that ERV activation in ATRT-SHH may also be modifiable (9).

Given that ATRT subgroups have distinct DNA methylation profiles and enhancer networks, efforts have been aimed at uncovering differential epigenetic regulation of immune pathways (26). These analyses have revealed that there is an overlap between differentially methylated regions (DMRs) and gene programs related to the innate immune response in ATRT-MYC. There is also an enrichment of interferon regulatory factors, inflammatory cytokines, and *HLA* transcription factor binding sites (TFBS) in enhancer regions in ATRT-MYC. TFBSs in ATRT-SHH and ATRT-TYR are enriched for neural genes (26). It is worth considering whether efforts aimed at pharmacological modulation of the epigenetic features that establish and maintain enhancer landscapes could allow for the rewiring of the TME in ATRT and other cancers.

### Subgroups of ependymoma

Ependymoma is another pediatric CNS malignancy that is divided into distinct subgroups, based on anatomic, molecular, and histological features. These tumors are recognized as arising from the posterior fossa (PF), supratentorial (ST), or spinal (SP) compartments. Pediatric ependymomas most commonly arise from the PF and ST compartments. PF group A ependymoma (PFA-EPN) is the dominant pediatric subgroup, while group B (PFB-EPN) primarily affects older children and adults (28). PFA-EPN has a paucity of somatic mutations and is largely driven by epigenetic alterations. EZHIP (previously CXorf67) is recognized as being overexpressed in PFA-EPN, functionally leading to PRC2 inhibition to produce global loss of H3K27me3, functionally similar to the H3K27M mutation in pediatric diffuse midline glioma (DMG) (29, 30). Consistent with this epigenetically driven biology, PFA-EPN has a low mutation burden, likely contributing to a relatively low level of neoantigen expression and an immune cold phenotype (31, 32).

Immunohistochemical profiling has demonstrated that PFA-EPN has relatively lower CD8+ T-cell infiltration compared to ST-EPN, another subgroup of childhood ependymoma (33). While lymphocyte infiltration is poor, myeloid cells were identified as infiltrating the tumor parenchyma (31, 32). Within this myeloid-dominated microenvironment, transcriptomic analyses by Griesinger et al. of PFA-EPN have shown enrichment in IL-6/STAT3 pathway signaling. The tumor-secreted IL-6 is associated with activating STAT3 in infiltrating myeloid cells, leading to increased IL-8 production and further polarization of myeloid cells to an immunosuppressive phenotype (34). Further work using single-cell and spatial analyses of PFA-EPN identified hypoxia myeloid cells, a subpopulation of myeloid cells. These cells showed features like myeloid-derived suppressor cells and also had similar IL-6/STAT3 pathway activation. Hypoxia myeloid cells on a spatial level are believed to further support an immunosuppressive tumor microenvironment by secreting IL-8 in both perivascular and necrotic tumor regions (35).

In contrast to PFA-EPN, childhood supratentorial ependymomas (ST-EPN) are associated with a more immune-active phenotype. A chromosomal fusion between ZFTA (formerly C11orf95) and RELA accounts for over 60% of supratentorial ependymomas, termed ST-EPN-ZFTA (36, 37). The resulting fusion oncoprotein has direct immunological effect through constitutive activation of the NF-κB pathway (36). Immunohistochemical analysis across 83 cases of ependymoma noted higher CD8+ T-cell density within the supratentorial compartment compared to posterior fossa tumors (33). An additional study showed tumor infiltrating CD4+ and CD8+ cells in ST-EPN-ZFTA had high expression of PD-1 but were functionally exhausted; both tumor and myeloid cells had expression of PD-L1 (38). This suggests an adaptive immune response is present but rendered inert by the tumor microenvironment. ST-EPN-YAP1, a less common pediatric supratentorial subtype, lacks high PD-L1 expression and carries a more favorable prognosis (38).

### Diffuse gliomas with oncogenic histone H3 K27M vs. G34R mutations

While several mutations can drive pediatric high-grade gliomas (pHGG), 80% of diffuse midline gliomas are defined by a lysine-to-methionine substitution in residue 27 of histone H3 (H3K27M) and 20% of diffuse hemispheric gliomas have a glycine-to-arginine/valine substitution in histone H3 at residue 34 (H3G34R/V) (39). Few other recurrent genetic mutations are present in these tumors, suggesting that non-genetic factors are central drivers of oncogenesis in these tumors. Indeed, H3K27M impairs H3K27 trimethylation and H3 G34R/V disrupts H3K36 trimethylation, leading to broad epigenetic dysregulation (39–41). Unlike ATRT where the same mutation leads to diverse immune microenvironments, Andrade et al. showed that histone-mutant pHGGs have a high infiltration of diverse myeloid cell populations and a low infiltration of effector cells, including T cells and NK cells. Additionally, compared to low-grade glioma, the microglia and macrophages of K27M-mutant tumors have higher levels of checkpoint molecules, such as HAVCR2 and lower expression of cytokines (42). Similar findings have been reported by others in K27M-mutant tumors (43). Additionally, Haase et al. demonstrated that H3.3 G34R mutations lead to impaired DNA repair responses and increased sensitivity to DNA damaging conditions. In this molecular background, DNA damage induces cGAS/STING signaling and consequently, IFN-β, suggesting that the G34R mutation modulates a cell’s capacity for immunogenicity (44). At baseline, it has also been suggested that the G34R mutation regulates the expression of immune pathways, such as the JAK-STAT pathway (45). Further studies comparing K27M and G34R/V mutant pHGGs will be needed to conclusively determine if and how different onco-histones can shape the TME.

## Overview of current immunotherapy approaches

Pediatric brain tumors can thus exploit several mechanisms to evade detection and lysis by the immune system. Numerous clinical trials are aimed at targeting these mechanisms as therapeutic vulnerabilities to reshape the tumor immune microenvironment to oppose tumor growth. Common approaches include immune checkpoint blockade, CAR T-cell therapy, dendritic cell vaccines, and viral immunotherapy. Recurrent barriers to efficacy that have emerged across immunotherapy modalities include an immunosuppressive TME, reduced cancer immunogenicity, low effector cell function, and CNS-specific barriers, including NirAEs, as summarized in **Figure 1**. The results and barriers of the described clinical trials are summarized in **Table 2**. 

**Table 2:** Summary of results of immunotherapy clinical trials relevant to pediatric brain tumors, with an emphasis on emerging barriers.

Immunotherapy modality	Summary of results	Barriers	Key references
Immune checkpoint blockade	Mixed results, with some failure to reach primary endpoints; well-tolerated	Low tumor immunogenicity; low lymphocytic infiltration; low target expression; persistent TAMs	(73, 80, 82) NCT03925246, NCT03493932, NCT03173950
CAR T cell therapy	Well-tolerated; feasible; evidence of immune response; can lead to stable disease and some durable responses	immunosuppressive microenvironment; antigen expression; CAR T cell persistence; cytokine/chemokine profile	(86–89) NCT03500991, NCT03638167, NCT04185038, NCT04196413
Vaccines	Tolerated and feasible; generation of specific immune response; evidence of durable favorable responses in several patients	Resection status; dendritic cell maturation; vaccine production time; MDSCs; low neoantigen diversity; low effector cell recruitment	(96–99, 102) NCT00107185, NCT04911621, NCT02840123
Oncolytic viruses	Increased infiltration of T cells; acceptable safety and tolerability; promising efficacy in adult glioma	immunosuppressive microenvironment; inconsistent effector cell infiltration	(107–110) NCT03178032, NCT00805376, NCT03043391, NCT02457845

### Checkpoint blockade

The first immune checkpoint, CTLA-4, was initially identified in 1987 and subsequent work in the 1990s established that full T-cell activation requires complex signaling interactions that can be hindered by immune checkpoint engagement. Allison and colleagues demonstrated that blockade of CTLA-4 could lead to rejection of established tumors *in vivo*, establishing the groundwork for what has become a revolutionary anticancer strategy (65, 66). Similarly, PD-1 was identified in 1992 and found to suppress T-cell activity upon engagement with its ligands PD-L1 and PD-L2, with subsequent work establishing blockade of the PD-1/PD-L1 axis as a cornerstone of cancer immunotherapy (67–69).To date, immune checkpoint blockade has had profound success in some highly aggressive adult cancers, including advanced melanoma and metastatic non-small-cell lung cancer (70, 71). Success is often seen in patients with mismatch repair deficiency (MMRD), high microsatellite instability (MSI), or high tumor mutational burden (TMB) (72). Below, we will review key clinical trials that have investigated checkpoint blockade strategies in pediatric brain tumors.

#### Summary of trials and barriers to efficacy

CheckMate-908 investigated use of the anti-PD-1 antibody nivolumab, alone or in combination with the anti-CTLA4 antibody ipilimumab, in 166 pediatric patients with high-grade CNS malignancies, including newly diagnosed DIPG and recurrent/progressive medulloblastoma, ependymoma, and high-grade glioma (NCT03130959). Nivolumab monotherapy and nivolumab in combination with ipilimumab had acceptable side effect profiles but failed to extend overall or progression free survival as combination therapy, relative to historical controls. Baseline PD-L1 expression was not predictive of response. Interestingly, one patient with a microsatellite instability-high (MSI-H) tumor had a partial response and a patient with a high tumor mutational burden (TMB) did not respond to therapy (73). TMB has been associated with response rate to checkpoint blockade previously, including in adult glioblastoma, and in pediatric patients (74–76).

For example, a clinical trial using nivolumab in pediatric solid tumors with mismatch repair deficiency (MMRD) or an elevated TMB found that of 5 glioblastoma patients included in the trial, 3 of them entered into a complete remission at follow-up (median of 37 months) (NCT02992964) (76). Although this study had a small sample size, it suggests that high mutational burden sensitizes to immune checkpoint blockade in pediatric brain tumors, as it does for adult tumors.

Additionally, several institutions have retrospectively reviewed individual instances of checkpoint blockade administration in their pediatric patients with CNS malignancies. At one institution, 31 DIPG patients were analyzed; 8 received reirradiation with nivolumab, 4 received reirradiation, and 19 were not given reirradiation. In this setting, there was a statistically significant prolongation of the median overall survival in patients who were reirradiated, compared to those who were not, however, there was no statistically significant additional benefit for survival with the addition of nivolumab (77). None of the patients appeared to have radiographic evidence of pseudoprogression, which has been positively correlated with immunotherapy response, though it is essential to correctly differentiate between pseudoprogression and true progression radiographically (78, 79).

Another institution retrospectively summarized their experience using nivolumab in recurrent or progressive pediatric brain tumors, including HGG, medulloblastoma, and ependymoma. Their cohort of patients had a low TMB and low or negative PD-L1 expression. Median time to progression was 13.7 weeks in PD-L1 positive patients and 4.2 weeks in PD-L1 negative patients, though this difference was not statistically significant (p=0.088). The GBM patient with the highest TMB had the longest radiographic response (80). This has been seen in other cases, too. In a case report of a pediatric patient with progressive GBM and mismatch repair deficiency (MMRD), nivolumab led to a 60% radiographic reduction of tumor size and a stable response 10 months after immunotherapy administration (81). Although these studies were not powered to draw definitive conclusions, it is worth speculating that limited efficacy could be due to low expression of PD-L1 or low immunogenicity of these tumors. Additionally, many of the tumors reviewed in these studies have low lymphocyte infiltration at baseline, suggesting that agents like nivolumab would be ineffective if the T cells are not trafficking to the tumor in the first place (42, 43).

Additionally, there are several trials that have assessed immunotherapy efficacy in patients with adult brain tumors. The REVOLUMAB trial is a phase II trial that investigated the efficacy of nivolumab in IDH-mutant high-grade gliomas that failed first line treatments (NCT03925246) (82). Although nivolumab was proven to be safe, the results of this trial were discouraging, with 90% of patients progressing during the trial period, and a median progression free survival (mPFS) rate of 1.84 months (95% confidence interval: 1.81–5.89 months). Of the 39 patients treated, tumor tissue was assessed in 6 and revealed an immunosuppressive environment in several of the samples, dominated by CD163+ myeloid cells (M2-like macrophages) and few infiltrating lymphocytes. Most samples had PD-1 positivity. Histological findings did not obviously associate with progression-free survival (PFS). Additionally, none of the patients had evidence of pseudoprogression, which is often seen in patients who go on to respond to immunotherapy (79, 82).

Another phase II trial explored the efficacy of nivolumab in patients over the age of 18 with recurrent CNS malignancies, including those with ependymoma, medulloblastoma, and ATRT (NCT03173950). Interim reports show that among 30 heavily pre-treated patients and 27 non-heavily pre-treated patients, 4 and 10 patients had stable disease for greater than 6 months, respectively (83, 84). Clinical and molecular features associated with disease response have not been reported.

Taken together, immune checkpoint blockade therapy has shown some promise in pediatric and adult CNS tumors, particularly in patients with elevated TMBs, however, the success has not been overwhelming. Given that the response is not absolute, even in patients with a high TMB, it is worth considering whether there are baseline features of the immune microenvironment that still must be addressed to overcome barriers to immune checkpoint blockade. Perhaps improved immune activation, including improved lymphocytic trafficking/infiltration, and a reshaping of the suppressive myeloid population would enhance responsiveness to immunotherapy. Indeed, an investigational study using a microdialysis catheter to assess cytokine changes in GBM patients treated with anti-PD-1 and anti-LAG-3 failed to see an appreciable increase in IFN-gamma secretion 5 days after the first dose of Nivolumab in any of the 13 patients (NCT03493932) (85).

### CAR-T cell therapy

Chimeric antigen receptor (CAR)-T cell therapy requires the expression of an engineered chimeric antigen receptor in autologous T cells ex vivo before infusion back into the patient. CARs are directed towards highly expressed antigens on malignant cells, providing selectivity towards cancer cells. Several trials have used CAR T cell therapy in pediatric patients with CNS malignancies with satisfactory safety and limited efficacy in phase 1 trials.

#### Summary of trials and barriers to efficacy

BrainChild01 uses a human epidermal growth factor receptor 2 (HER-2)-specific CAR delivered through locoregional infusion (NCT03500991). In a published interim report on 3 patients (2 metastatic ependymoma, and 1 anaplastic astrocytoma), 2 of 3 patients had progressive disease and 1 of the metastatic ependymoma patients had stable disease. CSF analysis for cytokine and chemokine alterations with treatment revealed elevations in the immunostimulatory interferon gamma (IFN-gamma) molecule, C-X-C motif ligand 10 (CXCL10), a lymphocyte chemoattractant, and the immunosuppressive chemokine, chemokine ligand 2 (CCL2), which recruits MDSCs. There was no clear correlation between cytokine and chemokine changes and clinical response. Interestingly, there was no evidence of CAR T cells in the CSF or periphery post-infusion in any patient (86).

BrainChild02 used an epidermal growth factor receptor (EGFR)-specific CAR delivered via locoregional infusion and has thus far treated 4 patients, 3 with HGG (2 K27-altered and 1 G34-altered) and 1 with ATRT (NCT03638167). 3 out of 4 patients had progressive disease, and there was no clear radiographic indication of pseudoprogression or immune response. CCL2 and CXCL10 levels were not assessed, but some patients had increases in IFN-gamma, IL-6 and IL-8 with CAR-T cell infusion (87).

BrainChild03 uses a B7-H3-specific CAR, and results have been published for 21 patients with a radiographic or histological diagnosis of DIPG (NCT04185038). mOS from the initial CAR infusion was 10.7 months, with a mOS of 19.8 months from diagnosis. 3 patients remained alive past 40 months, one of which had a histologically confirmed K27-altered DIPG. Matched pre- and post-CSF analysis revealed increases in CXCL10, granulocyte-macrophage colony-stimulating factor (GM-CSF), and IFN-gamma, indicating CAR-T activity, though the small sample size made it difficult to correlate molecular or immunological features with clinical response (88).

A CAR-T cell phase I trial against disialoganglioside (GD2) in biopsy-confirmed H3K27M+ DMG has also recently completed Arm A and has published results (NCT04196413) (89). 11 patients were treated, with 4 patients experiencing a major reduction in tumor bulk on imaging, and 1 complete responder at 30 months. It was demonstrated that the 8 patients who were defined as responders had elevated plasma IL-2 after CAR-T infusion and non-responders had higher levels of TGF-β and/or myeloid cell populations after CAR infusion. Plasma levels of CXCL10 decreased with progression. Additionally, depletion of peripheral levels of the CAR construct was temporally associated with progression (89).

There have also been efforts aimed at increasing the activity of GD2-directed CAR T cells. A phase I clinical trial has engineered GD2-directed CAR-T cells to express a constitutively active interleukin (IL)-7 receptor and has assessed safety and efficacy in a small number of pediatric patients with DMG or other CNS tumors with GD2 expression (NCT04099797). Of the 7 patients with DMG treated in the trial, 2 of them had partial responses. Of the 11 patients included in the trial, 9 of them had improvement in disease-related neurological deficits, and the improvement was more durable in patients receiving the GD2-directed CAR-T cells modified to express the constitutively active IL-7 receptor, as compared to those receiving GD2 CAR-T cells without constitutive activation of the IL-7 receptor (90).

Taken together, CAR-T cell therapy has shown promise in early phase I clinical trials, though it is difficult to ascertain efficacy or biological correlates associated with efficacy in such trials. However, published results from these trials show common trends associated with efficacy. For example, persistence of the CAR T cells in CSF and plasma appeared correlated with survival, as did a favorable cytokine/chemokine profile, characterized by high CXCL10 and low TGF-β. Additionally, expression of MDSC recruiting chemokines, such as CCL2, was evident in some patients, potentially indicating that remodeling the immunosuppressive microenvironment could be beneficial. Most of these trials also did not require histological target confirmation prior to therapy, raising the question of whether responders and non-responders segregate according to levels of antigen expression. Newer trials, such as BrainChild-04, will target multiple antigens simultaneously, thus aiming to circumvent potential resistance mechanisms or lack of uniform antigen expression (NCT05768880). Several of these limitations have also been considered barriers in CAR T cell trials aimed at adult CNS malignancies (91).

### Dendritic cell vaccines

Autologous dendritic cell vaccines load the patient’s own dendritic cells with immunogenic material ex vivo and then inject them back into the patient to stimulate a T-cell mediated antitumor response. Dendritic cells can be pulsed with several forms of antigenic material, including autologous or allogenic tumor lysate, viral mRNA, tumor mRNA, and more (92). Preclinical glioma models have had favorable responses to dendritic cell vaccines, as have select patients in published case reports (93–95). This led to the initiation of several clinical trials investigating the use of autologous dendritic cell vaccines in pediatric patients with central nervous system tumors, such as pHGG (including DMG), glioblastoma (GBM), ATRT, ependymoma, and medulloblastoma. Some of these clinical trials use autologous tumor material, such as tumor-lysate or tumor RNA (NCT00107185) (96, 97). Others have used dendritic cells pulsed with the mRNA of known tumor antigens, with viral mRNA, or with tumor cell line lysate (NCT03615404, NCT04911621) (98–100). Several of these were phase I trials performed in heavily pre-treated patients using small sample sizes, making it difficult to identify statistically significant improvements in PFS and overall survival (OS) within the group in comparison to historical controls.

#### Summary of trials and barriers to efficacy

While the results of trials using autologous dendritic cell vaccines are promising, they are not as efficacious in humans as they are in preclinical models. It is difficult to conclusively identify barriers to efficacy, given the small sample sizes in many studies, yet hypothesized barriers are manifold (101). For example, it is possible that the extent of resection prior to vaccine initiation, as well the use of immunotherapy as monotherapy or in combination with other agents may influence clinical response. Ardon et al. used a tumor-lysate loaded dendritic cell vaccine to assess efficacy in 45 children with central nervous system tumors, including 33 patients with pHGG, 5 patients with medulloblastoma/primitive neuroectodermal tumor (PNET), 4 with ependymoma, and 3 with ATRT. While not statistically significant, this study found an extension of PFS and OS in patients with complete resection prior to the initiation of immunotherapy as well as in patients who received both immunotherapy and chemotherapy. 6 relapsed HGG patients had an overall survival >24 months in this study (97). Others have also pointed to a correlation between the extent of residual disease and efficacy (102).

Additional efforts have evaluated the influence of dendritic cell maturation on efficacy. Ardon et al. treated cohorts using dendritic cells with different maturation schemes (97). Another study assessing tumor RNA-pulsed dendritic cell vaccines in patients with CNS tumors, did not mature the dendritic cells ex vivo before vaccination. This study included 2 patients with GBM and 3 patients with ependymoma. Only one of these patients had stable disease on the study; all others had progressive disease (96). Lasky et al. used a tumor lysate pulsed autologous dendritic cell vaccine in 3 HGG patients, 2 of whom had long-term survival exceeding 40 months (NCT00107185). This study did not fully mature the dendritic cells ex vivo (102).

Lasky et al. also found that feasibility in recurrent tumors was hindered by the production time of the vaccine, as several patients progressed between the time that it took to collect the tumor sample and produce the vaccine, making them ineligible for the study (102). Several studies also speculated on the role of the tumor immune microenvironment in driving efficacy. Olin et al. used autologous dendritic cells pulsed with an allogenic brain tumor cell line lysate in adult and pediatric patients with GBM, ependymoma, and medulloblastoma. They report a negative correlation between stable disease and circulating MDSCs and a positive correlation between natural killer cells and stable disease (103). Additionally, the one patient that had progressive disease in Lasky et al.’s cohort using an autologous dendritic cell vaccine pulsed with tumor cell lysate was found to have a modest increase in infiltrating lymphocytes prior to vaccination, but at recurrence (post-vaccination), had infiltrating macrophages, but not lymphocytes (102). Some have speculated whether tumors that have more modest responses to dendritic cell vaccines have lower quantity and diversity of neoantigens and others see the lack of shared tumor-associated proteins as a barrier to vaccine feasibility (97, 104).

Finally, though the numbers are small, two studies have pointed to higher efficacy in ATRT patients (97, 104). Although these studies did not separate their ATRT patients by the current subgroup classification defined by Johann et al. and others, it is worth wondering whether the ATRT patients that fared better were of the MYC or TYR subgroup that likely had higher lymphocytic infiltration at baseline, or from the SHH subgroup that, although usually has lower lymphocytic infiltration, also has lower immune checkpoint expression (22, 25, 26).

Taken together, it is suspected that dendritic cell vaccine efficacy is hampered by vaccine production limitations, use as monotherapy, subtotal resection prior to vaccine delivery, poor lymphocytic infiltration of activated cytotoxic lymphocytes, high proportion of MDSCs, and few, recurrent tumor-specific neoantigens to target, making personalized, autologous vaccines required. This suggests that there is room for optimization at the level of the tumor and its microenvironment, at the level of the autologous cellular product, and at the level of production.

### Viral immunotherapy

Oncolytic viral therapy is a two-pronged immunotherapy approach to target malignant cells. Therapy involves administration of a lytic virus engineered to have preferential virulence towards oncogenic cells over normal cells, leading to oncolysis; cancer cell destruction is highly immunogenic as tumor antigens are released into the microenvironment (105). Oncolytic viral therapy has had modest efficacy in pediatric and adult CNS malignancies, though clinical responses have not been absolute, leaving space for combinatorial approaches to improve efficacy.

#### Summary of trials and barriers to efficacy

The oncolytic adenovirus, DNX-2401, has been used in pediatric patients with DIPG and adults with recurrent glioma. In DNX-2401, the adenovirus genome is modified to have tropism for glioma cells and to replicate in cells with a defective Rb pathway (106). In 12 pediatric patients with DIPG, 9 patients had imaging that was consistent with reduced tumor size, and 8 patients had stable disease. The median survival was 17.8 months, and 1 patient continued to have PFS at 38 months (NCT03178032) (107). 11 of 12 patients also received radiation (median dose of 54 Gy) after initial DNX-2401 administration, and patients could also receive subsequent chemotherapy or reirradiation, making it difficult to attribute survival to DNX-2401 alone. At baseline, all patients had high myeloid and low lymphocyte infiltration, in agreement with previous reports on the TME of DIPG. In one patient, tissue from diagnosis, recurrence, and autopsy was available and revealed that the proportion of CD163+ M2-like macrophages and tumor infiltrating lymphocytes (TILs) were increased at relapse, while myeloid cell populations and regulatory T cells were reduced. At autopsy, CD163+ M2-like macrophages were elevated, while TILs were reduced. All patients were seropositive for adenovirus IgG antibodies, but patients with low antibody titers fared better, with an overall survival of 21.2 months, versus 12.5 months in patients with high titers (107). Though this was a small trial, it is worth speculating that the balance of cytolytic and immunosuppressive cells in the tumor microenvironment may be important for sustained responses to oncolytic viral therapy.

In adult patients, DNX-2401 had impressive efficacy in recurrent malignant glioma; 20% of patients survived 3 years after treatment, and 12% of patients had PFS at this time point (NCT00805376) (108). Responding patients had both radiographic and histological evidence of immune activation. Of the three complete responders, 2 were IDH1 wild-type and 1 was IDH1 mutant, and all had evidence of pseudo progression followed by >95% reduction in tumor size. All patients had later recurrence; in one of these patients, one of the recurrent sites only showed necrotic cells and no tumor cells, indicating a potential memory response (108). Biopsies of other patients also revealed masses that were predominantly necrotic with overwhelming inflammatory cell infiltration. Interestingly, DNX-2401 treatment was also associated with reduced expression of TIM-3, an immune checkpoint molecule. Finally, histological analysis revealed high infiltration of CD8+ T cells and the presence of immunogenic cell death after treatment (108). These two studies demonstrate that oncolytic viral therapy can induce potent immune responses in patients with CNS malignancies, with the adult glioma study suggesting that radiographic evidence of an immune response (i.e. pseudo-progression) may be an important indicator of response.

Another trial has used a recombinant polio and rhinovirus oncolytic virus, called PVSRIPO, in pediatric patients with recurrent HGG (NCT03043391) (109). CD155 is the receptor for the poliovirus, and it has high expression in brain tumors, allowing for oncolysis to be directed towards malignant cells. This therapy was delivered via convection enhanced delivery to children and young adults with heavily pre-treated HGG, ependymoma, ATRT, and medulloblastoma. 12 patients were treated but had poor efficacy with a mOS of 4.1 months, compared to a historical control of 5.6 months for standard treatment. This was a phase I study that was not powered to detect efficacy, and as such, the small sample size and the heterogenous group make it difficult to definitively determine the reasons for failure. Of the histological data reported, there were inconsistent increases in lymphocytic infiltration of the tumor following treatment. The authors speculated that immunosuppression from previous treatments may have limited responsiveness to therapy (109). Overall, the side effect profile was comparable to other immunotherapy treatments and included 3 grade 3 adverse events and 2, reversible, grade 4 events that were attributed to treatment. Swelling from therapy was managed with bevacizumab (109).

Finally, an oncolytic HSV-1 viral therapy, called G207, has been used in 12 pediatric patients with high grade glioma and was successful, with a mOS of 12.2 months and 4 patients remaining alive at 18 months post-treatment; patients who had seroconversion after treatment with G207 had clinically meaningful and statistically significant prolonged survival (NCT02457845) (110). Several patients had evidence of an inflammatory response, including pseudoprogression in one patient and polycystic degradation in 2 others. 4 patients had matched pre- and post-treatment biopsies, revealing an immune cold immune environment at baseline, and a profound increase in lymphocytic infiltration after treatment. Even a patient with severe lymphopenia prior to therapy had lymphocytic infiltration of the tumor bed after treatment with G207. Baseline NK cell count was positively correlated with survival. No grade 3 or 4 adverse events were reported. The authors suggest that the ability of G207 to convert an immune “cold” tumor to a “hot” one contributed to its impressive efficacy (110).

Overall, several oncolytic viral therapies have had impressive success in both pediatric and adult patients with aggressive CNS malignancies. Though the design of the studies and the heterogenous patient populations make it difficult to determine differences in the TME between responders and non-responders, it seems that seroconversion and low baseline antibody titers are important. Most studies reported a high infiltration of lymphocytes post-treatment and correlated low levels of checkpoint molecule expression positively with survival. Although not a universal theme, it seems that responders generally had a more potent immune response to therapy, suggesting that non-responders may benefit from an enhanced or sustained reshaping of the TME. Perhaps increasing effector cell function and reducing immunosuppressive myeloid cells may boost efficacy in non-responders.

### Combination approaches

Following safety establishment of immunotherapy as monotherapy, several clinical trials have assessed whether multiple immunotherapies can synergize to improve efficacy while maintaining safety. A study using an adenoviral vector containing the HSV tyrosine kinase gene in combination with valacyclovir, radiation therapy, and nivolumab found that OS was correlated with a baseline pro-inflammatory tumor microenvironment, including the presence of B cells, dendritic cells, and HLA-DR positive macrophages, and low expression of immune checkpoints, such as *PDCD1* (NCT03576612) (111).

Similarly, the CAPTIVE trial used DNX-2401 viral therapy in combination with the PD-1 inhibitor, pembrolizumab, in 49 patients with recurrent glioblastoma (NCT02798406). While the results were disappointing, with an objective response rate that did not differ from the prespecified control rate, there were several patients that had durable responses at 45, 48, and 60 months, despite a mOS of 12.5 months (112). Their ancillary analyses found that all patients who had an objective response had a baseline “intermediary” tumor microenvironment, characterized by a medium amount of immune cell infiltration and *PCDC1* expression, and low expression of non-target checkpoint molecules. These patients went on to have longer OS and a better clinical outcome (112).

Another trial using nivolumab, radiation therapy, and an indoleamine 2,3-dioxygenase 1 (IDO-1) inhibitor (with or without temozolomide) in newly diagnosed GBM also found a positive correlation between baseline and treatment-induced levels of early effector and naive CD8+ T cells in the periphery (NCT04047706) (113). There is also an investigational study using INO-5401, which contains 3 DNA plasmids for *WT1*, *PMSA*, and *hTERT*, and INO-9012, which contains a plasmid for IL-12, and cemiplimab, an anti-PD-1 antibody, in newly diagnosed GBM (114).

Taken together, there is overwhelming evidence that a baseline immunosuppressive tumor microenvironment has a deleterious impact on immunotherapy efficacy, especially for approaches that use immune checkpoint blockade. The several studies described above using immune checkpoint blockade monotherapy or combination therapy have demonstrated that responders have a proinflammatory environment, defined by increased lymphocytic infiltration and low expression of non-target checkpoint molecules (111–113). Overcoming barriers to effective immunotherapy should be aimed at reshaping the baseline tumor immune microenvironment.

## Leveraging epigenetic targeted agents to overcome barriers to immunotherapy

Though most completed clinical trials investigating immunotherapies in pediatric brain tumors have been phase I trials, and thus not powered to detect efficacy or biological correlates of efficacy, key barriers have still emerged. Across several immunotherapy platforms considered here, including CAR-T cells, autologous cell vaccines, immune checkpoint inhibitors, and oncolytic viruses, it is clear that barriers to efficacy include low immunogenicity, a baseline immunosuppressive TME, low effector function, and CNS-specific barriers, including NirAEs and potential immune cell trafficking issues. As such, immunotherapy approaches must balance a tight window between safety and efficacy. These barriers involve innate features of the tumor itself as well as the identity and activity of immune cells that comprise the tumor microenvironment. Importantly, both tumor immunogenicity and immune cell maturation and function are tightly regulated by epigenetic modifications (9, 115).

**Figure 3:**
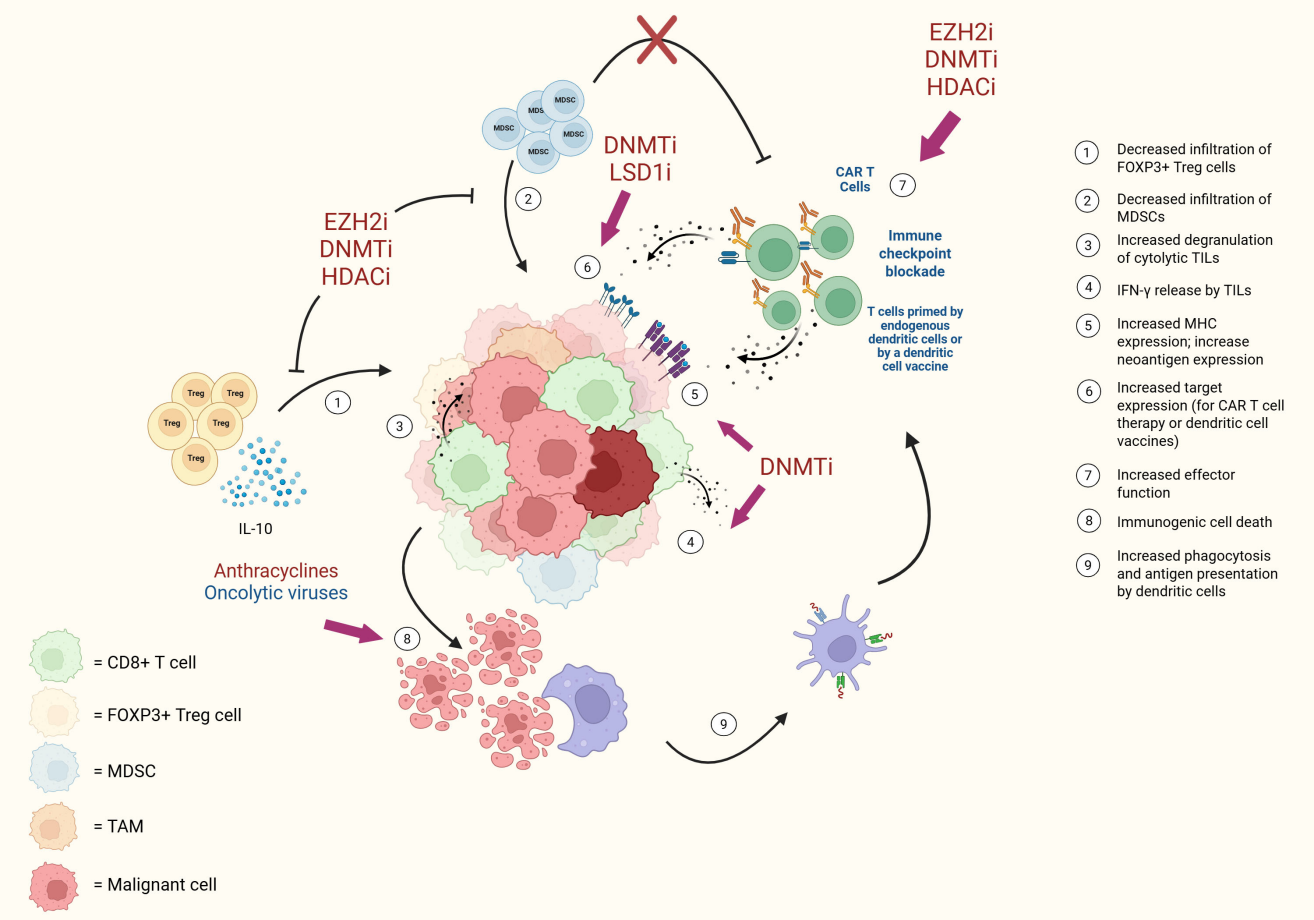
Epigenetic targeted therapies can be applied to overcome barriers to immunotherapy efficacy. EZH2, DNMT, and HDAC inhibitors have been shown to inhibit Treg cells (1) and MDSCs from infiltrating into the tumor (2). Inhibition of these immunosuppressive cells allows for increased release of degranulation of cytotoxic T cells (3); DNMT inhibitors also promote IFN-gamma release from cytotoxic T cells (4) and increased expression of neoantigens and MHC Class I expression (5). DNMT and LSD1 inhibitors can be leveraged to increase the expression of immunotherapy target molecules (6). EZH2, DNMT, and HDAC inhibitors can also be used to increase the activity and durability of effector cells stimulated by immunotherapy or effector cells engineered for immunotherapy (7). Finally, anthracyclines and oncolytic viruses can induce immunogenic cell death (ICD) (8) which ultimately increases antigen presentation by dendritic cells (9).

Outlined below are several approaches that use both novel and FDA-approved pharmacologic agents that target the epigenome to overcome the barriers to successful immunotherapy (**Figure 3**). This review will consider the use of several broad classes of epigenetic agents to target these barriers, including agents that modify DNA methylation, histone acetylation, and histone methylation. A comprehensive description of epigenetic abnormalities and the application of epigenetic-targeted agents to pediatric cancers has recently been published (116).

Most of the evidence supporting approaches combining epigenetic therapy with immunotherapy comes from adult tumors, rather than pediatric brain tumors, however, we hypothesize that similar findings will translate to the pediatric setting. Indeed, several of the reported findings have been consistent across different cancer diagnoses, perhaps suggesting that epigenetic modulation of the TME may be a general finding that can be applied to numerous cancer types. Findings in adult cancer models must be validated in immunocompetent models of pediatric CNS malignancies, however.

### DNA methylation

DNA methylation occurs at CpG sites (cytosine followed by a guanine in the 5’ to 3’ direction) in the genome. High deposition of this mark in promoter regions precludes transcription factor binding and leads to subsequent gene downregulation. DNA methyltransferase inhibitors (DNMTis), such as 5-Azacytidine (azacitidine) and 5-Aza-2’-deoxycytidine (decitabine) are small molecules that induce DNA hypomethylation (117).

### Histone acetylation

Acetylation is an activating chromatin mark that is deposited onto lysine residues in the histone tail by histone acetyltransferases (HATs). When added onto the tail of histones, the acetyl group relaxes the charge-based attraction between the positive lysine and the negative DNA backbone, creating space for the recruitment and binding of transcriptional machinery, such as transcriptional activators. This mark is also essential for the establishment of enhancers and is often dysregulated in cancer. Histone acetylation is removed by histone deacetylases (HDACs), and several HDAC inhibitors are used clinically, including vorinostat, panobinostat, and entinostat (118). Unfortunately, panobinostat had its FDA approval withdrawn due to the failure of the producing company to complete postmarketing clinical trials (119).

### Histone methylation

Methylation is another histone mark that is typically deposited on lysine and arginine residues in the tails of histone H3. Though histone acetylation is always an activating mark, histone methylation can activate or repress gene expression, depending on the residue that is being modified. Additionally, histones can be marked with mono-, di-, or tri-methylation (120). Distinct enzymatic complexes are responsible for the deposition of histone methylation. For example, polycomb repressive complex 2 (PRC2) contains the histone methyltransferase subunit enhancer of zeste homolog 2 (EZH2), which methylates H3K27 (121). Methylation of H3K9 is mediated by euchromatic histone lysine methyltransferase 2 (EHMT2), which is also known as G9a and suppressor of variegation 3–9 homolog 1 and 2 (SUV39H1/2) (122). Histone demethylation is regulated by separate enzymatic complexes, including lysine specific histone demethylase 1A (LSD1) which demethylates H3K4me1/2 and H3K9me1/2 (123).

### Overcoming reduced immunogenicity

Several immunotherapy approaches rely on the expression of specific targets (i.e. CAR T cells, some dendritic cell vaccines, etc.), however cancer cells can express these target molecules lowly or unevenly or downregulate them with environmental pressures. Below, we consider how pharmacologic modulation of the epigenome can enhance the immunogenicity of the tumor immune microenvironment by several mechanisms, including increasing viral mimicry and neoantigen expression.

#### Viral mimicry

Some agents, such as DNA hypomethylating agents, can increase the expression of cytosolic double stranded RNA (dsRNA) in cancer, and this can lead to downstream upregulation of interferons and interferon stimulated genes (ISGs). This causes the cancer cell to appear as if it is infected with a virus (“viral mimicry”), thus stimulating enhanced recognition by the immune system (9, 124).

In some cases, the molecular features of the tumor poise a cell towards a viral mimicry response. In H3K27M-mutant DMG, for example, the onco-histone leads to increased H3K27ac over repetitive elements, and increased expression of repetitive elements and endogenous retroviruses (ERVs) relative to isogenic histone wild-type controls. ERV and repetitive element expression can be further heightened in this context by using the combination of an HDACi and a DNMTi, leading to viral mimicry (11). The combination of an HDACi and a DNMTi has been suggested in other tumors as well (10).

Pharmacologic manipulation of other aspects of the epigenome can also trigger viral mimicry. For example, LSD1 depletion in poorly immunogenic melanoma led to dsRNA stress, an IFN response, increased T cell infiltration, and enhanced responsiveness to anti-PD-1 therapy (125). A follow up study identified an upregulation of TGF-β following LSD1 knockout, and adding TGF-β blockade to the combination of LSD1 ablation and anti-PD-1 therapy enhanced T cell cytotoxicity and led to eradication of tumors *in vivo* (126). Additionally, shRNA knockdown of LSD1 in glioblastoma cells resulted in a transcriptional upregulation of the “immune response” pathway, but not dsRNA stress response genes, indicating potential differences in response to LSD1 inhibition in different tissue types (127).

SETDB1 knockout in several adult cancer models has been shown to induce expression of transposable elements and interferon-stimulated genes *in vitro* and *in vivo*, as well as increased infiltration of CD4+ and CD8+ T cells and NK cells *in vivo* (128–131). Additionally, knocking down ZNF638 in glioblastoma cells decreased H3K9me3 while increasing retroelement expression and antiviral dsRNA sensing pathway signaling (132).

Additionally, pharmacologic inhibition or knock down of components that regulate histone methylation, such as EZH2, G9a, or SUV39H1 synergizes with decitabine to induce ERV and viral response genes in several cancers, including colon cancer, leukemia, ovarian cancer, and bladder cancer (133–136). SUV39H1 KD in AML cells also induced retrotransposon expression and IFN pathway activation, while destabilizing G9a/GLP, and combined UNC0638 and 5-AZA treatment of AML cells synergistically upregulated IFN pathway genes (137).

Epigenetic induction of viral mimicry may synergize with immunotherapy by increasing the infiltration and activity of key effector cells. The “viral mimicry” response has been demonstrated across several cancer types, including both adult and pediatric tumors, suggesting that it might be a principle that could be applied broadly to pediatric brain tumors.

#### Immunogenic cell death and viral mimicry

Immunogenic cell death (ICD) can enhance the immune visibility of cancer cells by increasing phagocytosis by dendritic cells and subsequent presentation of tumor-specific antigens to T cells (138). Phagocytosis is stimulated by damage-associated molecular patterns (DAMPs), such as presentation of calreticulin on the surface of tumor cells, and the release of soluble factors, such as high mobility group box 1 (HMGB1), leading to dendritic cell activation (139, 140). Interestingly, the chemotherapy agent doxorubicin has been shown to induce immunogenic cell death, as evidenced by enhanced HMGB1 secretion, increased phagocytosis by macrophages and IFN-gamma-producing T cells, and by decreasing Treg cell populations (141).

Like other epigenetic modifiers discussed above, anthracyclines have also been shown to induce a ‘viral mimicry’ response, leading to activation of the type I interferon response and upregulation of immune recruiting cytokines and chemokines such as CXCL10. Interestingly, neutralizing antibodies targeting interferons almost completely block the efficacy of doxorubicin *in vivo* and treatment with RNase prevents doxorubicin-induced upregulation of CXCL10 (142). It is possible that the epigenetic mechanisms of anthracyclines could be leveraged to augment immunotherapy, suggesting an exciting new avenue of research.

#### Increasing neoantigen levels

Many pediatric CNS malignancies have low expression of neoantigens and cancer testis antigens at baseline, and this impedes immune surveillance. In the context of immunotherapy, low neoantigen expression translates into fewer targets for CAR-T cells and dendritic cell vaccines. Additionally, low expression of select targets could lead to increased susceptibility to immunotherapy resistance.

There are several promising approaches to increase neoantigen diversity by using experimental compounds used preclinically. LSD1, along with HDAC1/2 and the scaffold protein CoREST, is a part of the CoREST epigenetic repressor complex. Corin, a bifunctional drug targeting the HDAC and LSD1 components of this complex, was found to disrupt the CoREST complex-mediated splicing program in melanoma cells, leading to immunogenic neoantigen induction, which enhanced the response of immunocompetent mice with melanoma to anti-PD-1 therapy (143, 144). Although follow up experiments are warranted, similar RNA splicing differences were found in ATRT cells treated with corin; treatment of ATRT (and potentially other immune cold pediatric brain tumors) with corin may also increase neoantigen expression and tumor immunity (144).

Even in the absence of high neoantigen expression, epigenetic agents can be applied to enhance immunotherapy efficacy. LSD1 inhibition by the reversible scaffolding inhibitor SP-2509 increased FAS expression in *TP53* wild-type neuroblastoma cells, resulting in enhanced killing of cells with low antigen expression by CAR T cells *in vitro* through the FAS-FASL axis (145). Although this strategy may be specifically tailored to neuroblastoma due to its increased expression of LSD1, epigenetically activating FAS may be a useful technique to sensitize tumors expressing low levels of antigens to CAR T therapy (145).

Genomic regions in mouse melanoma and mouse Lewis lung carcinoma (LLC) containing SETDB1-dependent H3K9me3 were enriched for transposable elements (TEs) in the long terminal repeat (LTR) family, as well as segmental duplications, which were enriched for genes involved in immune-related processes. Many of these loci became activated upon *Setdb1* knockout (KO), including TEs that encoded putative MHC-1 peptides recognizable by T cells (128). A similar finding was seen upon SETDB1 KO in human BRAF-mutant melanoma (128).

Additionally, upregulation of cancer testis antigens (CTAs), such as NY-ESO-1, the melanoma antigen gene (MAGE) antigens, and PRAME, with hypomethylating and/or hyperacetylating treatment has been shown several times, including in pediatric brain cancers, such as H3K27M-mutant DMG and ATRT (146–151). Demonstrations in neuroblastoma cell lines showed that upregulation of the CTAs MAGE-A1, MAGE-A3, and NY-ESO-1 by decitabine can enhance killing by cytotoxic T lymphocytes previously exposed to peptide-specific dendritic cells in co-culture with IFN-gamma (146). This led to the initiation of a phase I clinical trial that used an allogenic dendritic cell vaccine targeted against NY-ESO-1, MAGE-A1, and MAGE-A3 in decitabine treated patients with relapsed or refractory solid tumors (NCT01241162) (12).

#### Major histocompatibility complex expression

Epigenetic targeted therapy can also enhance immunotherapy by increasing the expression of MHC proteins in cancer cells. Decitabine, for example, has been shown to upregulate both neoantigens and MHC expression in several cancer types *in vitro*, like sarcoma, ovarian cancer, adult glioblastoma, and several pediatric brain cancers, including H3K27M-mutant DIPG (147, 152–155). In cases where resulting T cell effector function was assessed, this was met with enhanced T cell responses (152, 153). Decitabine has been shown *in vivo* to enhance the effect of oncolytic viral therapy in a syngeneic triple-negative breast cancer model, likely due to enhanced immunogenicity and increased MHC Class I expression (156).

Additionally, mouse GBM cells and human glioma stem cells (GSCs) exhibited decreased PD-L1 and increased MHC-1 expression upon G9a KD *in vitro* (157). Co-culture of GSCs with CD8+ T cells isolated from human PBMCs demonstrated increased cytotoxicity with G9a KD in GSCs (157). The authors demonstrated that the Notch1 pathway plays a role in G9a regulation of the immune response, as Notch1 inhibition reversed changes in MHC-1 and PD-L1 expression, as well as increased cytotoxicity of CD8+ T cells in co-culture, induced by G9a overexpression (157).

#### Overcoming an immunosuppressive microenvironment

Efforts in pediatric brain tumors and in adult tumors resistant to immunotherapies can seek to leverage small molecules that target the epigenome to create a tumor microenvironment more favorable to immunotherapy. Below, we will outline ways in which pharmacological modulation of the epigenome can be leveraged to reshape the TME, by reprogramming MDSCs, macrophages, regulatory T cells, and effector cells towards an anti-cancer phenotype.

#### MDSCs

There is compelling preclinical and clinical evidence in adult tumors and some pediatric tumors that immune checkpoint blockade efficacy is hampered by an immunosuppressive environment. Impressively, several preclinical studies have provided evidence that one population of immunosuppressive cells, MDSCs, can be modulated pharmacologically, particularly with histone deacetylase inhibitors (HDACi). HDAC inhibitors can increase acetylation in both nuclear histones and cytosolic proteins; the targeting of histone proteins can lead to hyperacetylation and transcriptional modulation (158). In mice with established tumors of 4T1, a spontaneous and metastatic murine mammary cancer, dual treatment with anti-PD-1 and anti-CTLA-4 antibodies failed to control local tumor growth or the development of metastatic lesions. Combination of dual checkpoint inhibition with the HDACi, entinostat, and the DNA methyltransferase inhibitor, 5-azacytidine, led to 80% survival at 100 days post-tumor implantation with no metastatic lesions. This synergy was attributed to a remodeling of the immune microenvironment. Indeed, while treatment with 5-azacytidine and entinostat didn’t enhance CD8+ T cell infiltration in this model, it did reduce tumor infiltrating FOXP3+ Treg cells and peripheral and tumor infiltrating granulocytic MDSCs (159). Similarly, experiments in aggressive breast cancer and pancreatic cancer mouse models combining entinostat and anti-PD-1 and anti-CTLA-4 antibodies also demonstrate a reduction in TME MDSCs; although, in this model, there was also an increase in infiltrating cytotoxic lymphocytes (13). There is evidence that decitabine can also reduce the MDSC population in mouse models, which is hypothesized to enhance immunotherapy efficacy (156).

Additionally, the MDSC population can be reshaped by modulating histone methylation. Knocking down ZNF638, a DNA binding protein needed to recruit the human silencing hub (HUSH) complex, which recruits SETDB1, in an immunocompetent glioblastoma mouse model treated with anti-PD-L1 resulted in a shift from monocytic MDSCs to the less immunosuppressive granulocytic MDSCs in the tumor microenvironment (132). Additionally, KD of G9a in an immunocompetent glioblastoma mouse model led to decreased percentages of monocytic MDSCs and granulocyte-like MDSCs in the tumor microenvironment (157).

There is clear preclinical evidence suggesting that epigenetic therapies can effectively remodel the tumor microenvironment by decreasing the prevalence and activity of immunosuppressive MDSC populations. This has led to the initiation of clinical trials aimed at assessing whether this can enhance checkpoint blockade efficacy in patients. A phase I clinical trial investigating the use of entinostat, nivolumab, and ipilimumab in the treatment of aggressive HER2 negative breast cancer has shown promising results and demonstrates modulation of the peripheral MDSC population (14). Additionally, the INFORM2 trial (NCT03838042) is investigating the combination of entinostat and nivolumab in pediatric patients with solid tumors, including CNS malignancies. While the clinical trial is exploring immune infiltration as a biomarker for therapy success, it will be interesting to see whether and how this treatment modulates both MDSCs and effector T cells in the tumor immune microenvironment (160). Findings from this study may help to address whether epigenetic remodeling of the MDSC population can be translated to pediatric tumors.

#### Macrophages

In addition to MDSCs, macrophages also play a critical role in maintaining an immunosuppressive TME. The TAM population is comprised of both resident tissue macrophages (microglia) as well as recruited peripheral monocytes that differentiate locally into macrophages (161, 162). The recruited TAMs can be further programmed into pro-tumor or anti-tumor macrophages based on cytokines and signals produced by the cancer cells in the TME. Pro-tumor macrophages secrete distinct factors that can either directly support tumor growth or suppress other effector cells (163). Importantly, though macrophages have been previously classified as pro-tumor (M2) or anti-tumor (M1), phenotypically similar macrophages can perform distinct and opposing roles in different cancer contexts (162).

Pharmacological modulation of macrophage populations should be aimed at enhancing macrophage effector function (i.e. phagocytosis) and reducing pro-tumor macrophages (162). Epigenetic modifying small molecule drugs, such as histone deacetylase (HDAC) inhibitors, may end up being leveraged towards this end. For example, there is evidence that co-culture of the GBM cell line, U87, with THP-1 cells differentiated into macrophages leads to a decrease in TGF-β signaling and an increase in TNF-α signaling when treated with HDAC inhibitors, suggesting a shift towards an anti-tumor phenotype (164).

Reshaping the TAM population must be approached very carefully, however, as several epigenetic targeted agents, including HDAC inhibitors, are also being investigated for their ability to dampen macrophage activation in autoimmune or inflammatory conditions (165–167). Thus, just as macrophages have distinct pro- or anti-tumor functionality in tissue contexts, HDAC inhibitors may also have opposing effects on macrophages in different disease contexts such as autoimmune diseases and cancer (168–170). Further work will be necessary to clarify how epigenetic manipulation can be utilized to shape a macrophage population that can target cancer cells and promote effector cell activity.

#### Treg cells

Regulatory T cells are CD4+/CD25+/FOXP3+ T cells that play a critical role in attenuating immune responses and supporting an immunosuppressive environment in the TME (171). Enhancer of zeste homolog 2 (EZH2), a component of PRC2, is critical for Treg cell functionality (172). Indeed, EZH2 plays an essential role in establishing Treg-specific transcriptional programs downstream of CD28 signaling, and deletion of EZH2 impairs Treg cell activity and potentiates autoimmune responses in mice (172).

It has been shown in mice challenged with bladder cancer that antitumor immunity is enhanced in mice with Treg cells deficient in EZH2, leading to markedly suppressed tumor growth rates. This is accompanied by a pronounced increase in intratumoral CD8+ T cells positive for IFN-gamma or granzyme (173). Similar results were also shown in colorectal cancer (174).

In patients, treatment with the anti-CTLA-4 monoclonal antibody, ipilimumab, led to elevated EZH2 expression in peripheral Tregs and in CD4+ and CD8+ T cells (173). To assess if this was a therapeutic vulnerability, tumor-bearing mice challenged with cancer were co-treated with anti-CTLA-4 therapy and an EZH2 inhibitor. Combination therapy led to a marked suppression of tumor growth, which was accompanied by a significant decrease in intratumoral FoxP3+ cells and an increase in IFN-gamma+ CD8+ T cells, suggesting that combination treatment decreases infiltrating Treg cells and increases cytotoxic effector cell infiltration (173). Given that PRC2 activity is critical for Treg biology, it is likely that these findings would translate to pediatric brain tumors.

### Overcoming low effector cell function

All immunotherapies currently available rely on the activity of effector cells, such as T cells and NK cells. The effector function of these cells can be hindered by immune checkpoint molecules, low expression of activating molecules, and the transition towards an exhausted phenotype. Epigenetic agents can overcome these barriers.

#### T cells

In addition to modulating Treg cell function, it has also been shown that EZH2 inhibition can reshape effector T cell populations. Co-culture of the leukemia cell line, NALM6, with naive CD4+ and CD8+ T cells in the presence of a bispecific T-cell engager (BiTE) increases target cell lysis at lower effector-to-target cell ratios when cultured in the presence of an EZH2i (173). Additionally, there is evidence that CAR T cells manufactured from mice pre-treated with an EZH2 inhibitor have more durable responses, likely due to a reduced exhausted T cell population (fewer PD1+ cells), and a persistent memory T cell population (175). This promising preclinical evidence has led to the initiation of 2 clinical trials investigating the combination of tazemetostat with CAR T cell therapy (NCT05934838) and tazemetostat in combination with mosunetuzumab, a BiTE (NCT05994235). Additionally, the combination of EZH2 inhibitors and checkpoint inhibitors is also being investigated clinically in pediatric patients, due to the critical role of EZH2 in T cell biology. The TAZNI trial is investigating the combination of tazemetostat, nivolumab, and ipilimumab in pediatric patients with SMARCB1 or SMARCA4 deficiency, including patients with ATRT (NCT05407441) (176).

Decitabine pre-treatment of CAR T cells also significantly reduces T cell exhaustion, as evidenced by down-regulation of key checkpoints, such as *CTLA-4* and *LAG3. In vitro* co-culture experiments with decitabine-treated CAR T cells were more efficient at killing target cells, had higher evidence of degranulation and proliferation, had lower expression of markers of exhaustion (ex: PD-1), extended memory, and higher secretion of cytokines such as IFN-gamma. This approach had promising *in vivo* efficacy in immunodeficient mice bearing acute lymphoblastic leukemia (ALL) or non-Hodgkin’s lymphoma (NHL) (177). A clinical trial investigating CD19/CD20 CAR T cells in relapsed/refractory diffuse large B cell lymphoma in patients who had a pre-treatment regimen that included decitabine had increased survival compared to outcomes of a similar trial by the same investigators without decitabine (NCT03196830). The CRR and OR improved from 70.3% and 34.5% respectively to 90.9% and 63.6% with decitabine pre-treatment (178).

The combination of decitabine and CAR T cells has also been assessed in mouse models of Group 3 medulloblastoma and ependymoma (179). Using monospecific (EPHA2 for medulloblastoma or HER-2 for ependymoma) and tri-valent (EPHA2, HER-2, and IL13Rα2), Donovan et al. showed that the hypomethylating therapy, azacitidine, and CAR T cell therapy have impressive efficacy in combination. In medulloblastoma, for example, 100% of mice treated with azacitidine and EPHA2 CAR T cells were still alive beyond 200 days, whereas monotherapy-treated and control mice began to succumb to tumor-related progression at or prior to 100 days. The role of azacitidine in prolonging survival was not fully elucidated. While it was shown to increase the expression of EPHA2 in medulloblastoma, it was not evaluated whether azacitidine also modulated the tumor microenvironment or directly impacted the efficacy and durability of the CAR T cells (179).

Finally, decitabine and checkpoint blockade therapy has been shown to be a powerful combination preclinically and has led to the initiation of several clinical trials in adults that have had promising results in relapsed and refractory leukemia (NCT02996474) and locally advanced, HER2-negative breast cancer (NCT02957968) (180–182). In patients with breast cancer, decitabine treatment decreased MDSCs, increased TILs, and increased PD-L1 expression. This suggests that combination treatment can lead to enhanced effector cell function and that the immune checkpoint blockade may be needed to address enhanced checkpoint expression from decitabine (180).

Unfortunately, in one study that assessed the combination of decitabine and checkpoint blockade in syngeneic orthotopic mouse models of pediatric CNS malignancies, including medulloblastoma, ATRT, and H3K27M-mutant DMG, the combination was disappointing in orthotopic models, but promising in flank models (155). In contrast to other studies, this study did not find that decitabine increased PD-L1 expression, thus using decitabine would have only modulated the TME, without directly upregulating target expression. Additionally, another study found that while PD-1 checkpoint blockade had disappointing efficacy as monotherapy, dual blockade of PD-1 and CFSR1, which regulates myeloid cell survival, meaningfully extended survival in an immunocompetent mouse model of DMG (42). Given that there is strong evidence that modulation of histone modifications can effectively reshape myeloid-derived populations in several *in vitro* and *in vivo* contexts, perhaps PD-1 blockade in DMG will have greater efficacy in combination with epigenetic-targeted agents, other than decitabine. It is unclear how well the DNA methylation landscape of these syngeneic models match true pediatric CNS malignancies, or if the low dose of decitabine used in this study was able to reach sufficient concentrations in the CNS; these limitations make it difficult to rule out the potential of combinatorial immunotherapy approaches for pediatric patients with CNS malignancies.

In addition to modulating MDSC populations, altering histone methylation can also enhance T cell effector function. Knock-out (KO) of *Setdb1* synergized with anti-PD-L1 therapy in a murine syngeneic orthotopic ovarian cancer model to reduce tumor burden and increase CD8+ T cell infiltration (129). Additionally, knocking down ZNF638 in GBM cells increased PD-L1 expression *in vitro*, suggesting the possibility that manipulation of H3K9 methylation may synergize with anti-PD-L1 therapy (132). In line with this expectation, an immunocompetent syngeneic orthotopic GBM murine model with a ZNF638 knockdown treated with anti-PD-L1 survived longer than control, ZNF638-KD alone, or anti-PD-L1 alone. Additionally, this model had a lower reduction of H3K9me3, and this led to several consequences that increased CD8+ TILs and NK cell infiltration, including enhanced ERV expression, increased inflammatory cytokine expression, increased dsRNA sensing pathway activation, increased CD8+ TILs, and increased NK cell infiltration (132).

G9a KO in mouse hepatoma cells directly led to an increase in chemokine expression, with the greatest enrichment in CXCL10, which exhibited a loss of H3K9me2 at its promoter. This finding was consistent with pharmacological inhibition of H3K9me1/2 by UNC0642, an inhibitor of G9a and G9a-like protein (GLP), which forms a complex with G9a to catalyze H3K9me1/2, as well (183). In a transwell migration assay, CD8+ T cells exhibited enhanced migration toward hepatoma cells treated with UNC0642 *in vitro*. This was attributed to increased CXCL10, as the effect was blocked by a CXCL10-neutralizing antibody (183). G9a knockout or treatment with UNC0642 in an immunocompetent mouse hepatoma model increased infiltration of CD4+, CD8+, and regulatory T cells and reduced tumor growth, and depletion of CD8+ T cells reduced the tumor growth inhibition seen with G9a KO (183). Although no change in PD-L1 expression with G9a KO or UNC0642 treatment was seen, UNC0642 and anti-PD-1 treatment synergized in inhibiting tumor growth (183).

Treatment of pancreatic ductal adenocarcinoma (PDAC) cells with the dual G9a and DNMT inhibitor, CM272, led to expression of genes in categories including antigen processing and presentation via MHC Class I, type I interferon signaling, and processes related to T cell mediated immunity (184). Pre-treating PDAC cells with CM272 increased induction of immune response-related genes upon IFN-γ exposure (184). Combination treatment with CM272 and anti-PD-L1 antibodies in immunocompetent mice with an immune cold, immune checkpoint inhibitor-resistant murine PDAC tumor led to significant tumor regression, as well as increased CD4+ and CD8+ T cell infiltration (184).

Segovia et al. found that CM272 had a more profound effect on bladder cancer cells without *PIK3CA* mutations, which have normal EZH2 levels (134). In an *in vivo* murine bladder cancer model, combination of CM272 and cisplatin increased interferon and inflammatory response genes, as well as CD8+ T and NK cell infiltration. Treatment of bladder cancer cells with CM272 *in vitro* increased PD-L1 expression, and mice treated with CM272 + anti-PD-L1 had increased infiltration of CD3+, CD8+, and NK cells, as well as reduced primary tumor or metastatic disease (134).

T cell effector function can also be increased by altering cytokine and chemokine production and secretion. Seier et al. found that treating *MYCN*-amplified neuroblastoma cells with the G9a/GLP inhibitor, UNC-0638, and the EZH2 inhibitor, EPZ011989, enhanced interferon response to IFN-γ-inducible genes, including Th1-type chemokines, genes involved in antigen presentation, pattern recognition receptors, and immune checkpoints (185). Co-culture of activated (CD69+, IFN-γ+) T cells with *MYCN*-amplified neuroblastoma cells pretreated with UNC-0638 and EPZ011989 resulted in increased *CXCL9/10* expression and increased MHC class I surface expression on the neuroblastoma cells, indicating that these drugs may help to increase the weak IFN signaling present in the tumor’s cold tumor microenvironment (185).

Treatment of IFN-γ stimulated *Trp53-/-* murine ovarian cancer cells with the G9a/GLP inhibitor, UNC0642, and the EZH2 inhibitor, UNC19999, resulted an induction of CXCL10 mRNA and protein that was greater than either drug alone; an even greater increase was seen with the dual G9a/EZH2 inhibitor HKMTI-1-005 (186). HKMTI-1–005 was also confirmed to upregulate *CXCL10* mRNA in human ovarian high-grade serious carcinoma (HGSC) cell lines, as well as ascites-derived primary cells from HGSC patients (186).

Treatment of primary Group 3 medulloblastoma cells with the PRUNE-1 inhibitor AA7.1 and the reversible scaffolding LSD1 inhibitor SP-2577 led to an upregulation of genes involved in immune response pathways, including antigen presentation; the simultaneous decrease in TGF-β pathway activation and the known role TGF-β plays in immune suppression indicates that TGF-β may play an intermediary role in this response (187).

Modulating histone methylation within effector cells has also been found to be effective in reducing tumor growth (188). *Suv39h1* KO mice challenged with a murine melanoma model exhibited increased tumor rejection following anti-PD-1 treatment; this effect was recapitulated in mice with *Suv39h1* KO in T cells only, indicating that Suv39h1 plays a specific role in this population (188). Additionally, *Suv39h1* KO mice treated with anti-PD-1 had increased CD8+ TILs (188). Additionally, *SUV39H1*-disruption in CAR T cells improved early CAR T cell expansion and long-term persistence, protecting against multiple NALM6 tumor rechallenges and decreasing expression of inhibitory receptors (189).

Inhibitors that target the BET family proteins, which are known as “readers” of histone acetylation were prioritized by the 2020 ACCELERATE Paediatric Strategy Forum, with the goal of developing BET inhibitors with improved blood-brain barrier penetrance, a broader therapeutic index, and second generation BET inhibitors that selectively inhibit the BDII bromodomain (190–192). There is some evidence that these inhibitors also have immunomodulatory effects. JQ1, a brain-penetrant BET inhibitor, downregulated interferon-stimulated genes and PD-L1 in human GBM-derived sphere-lines, adherent GBM cell lines, and orthotopic GBM xenografts in mice (193). The decrease in PD-L1 expression indicates the possibility of synergy with immune checkpoint blockade, as has been seen *in vivo* in Myc-driven B cell lymphoma, although the reduction in interferon-stimulated gene expression potentially contradicts this and will need to be investigated further (193, 194). JQ1 treatment in combination with CBL0137, was found to upregulate expression of interferon response genes and MHC class I genes in DMG cells (195). CBL0137 is a brain-penetrant inhibitor of the histone chaperone facilitates chromatin transcription (FACT) that is currently in phase 1/2 clinical trial for patients with relapsed or refractory solid tumors including DMG (NCT04870944) (196). Combination treatment in an immunocompetent *in vivo* model resulted in decreased tumor burden and the top upregulated gene was *Ifi27* (195).

#### Natural killer cells

Some pediatric CNS tumors, such as DMG, are already sensitive to NK-cell mediated killing, likely due to down-regulation of MHC molecules (43). However, epigenetic-targeted therapies can also augment NK cell activity for tumors that have low NK cell activity. For example, treatment with the HDACi, entinostat, has been shown to increase the expression of ligands for Natural Killer Group 2 Membrane D (NKG2D) and the secretion of cytokines, such as CCL3, that recruit NK cells in a urothelial carcinoma model. Upregulation of these factors occurs downstream of entinostat-induced epigenetic modulation. This led to a marked increase in the infiltration of NK cells in a syngeneic bladder cancer mouse model (197).

Additionally, treatment of DMG cells with irreversible catalytic LSD1 inhibitors induced upregulation of gene sets related to the immune system, including individual genes such as SLAMF7, MICB, and ULBP-4, which are innate immune receptors that are involved in NK cell signaling. NK cell, but not T cell, lysis of these treated cells was increased compared to control-treated cells (127). Furthermore, mice with orthotopic DMG tumors treated with intraperitoneal GSK-LSD1 (one of the irreversible catalytic LSD1 inhibitors) and intracranial human *ex vivo* expanded NK cells had a greater reduction in tumor burden than vehicle or single agent treatment (127).

Thus, there are multiple ways to enhance effector function in immunotherapy by modulating the epigenome, affording clinicians and researchers with several options when aiming to enhance immunotherapy efficacy. Importantly, however, decitabine, some LSD1 inhibitors, and the newest G9a/GLP inhibitors are brain-penetrant, however, not all epigenetic agents are, meaning that investigators must be cautious when considering which agents to use (198–200). Application of a brain-penetrant epigenetic modulator may be more effective when trying to modulate and sustain responses at the tumor site, however, pre-treatment of peripheral immune cells may not require brain-penetrant therapy, unless that therapy also activates the tumor cells, themselves.

### CNS-specific considerations

In addition to immune-related and tumor-related barriers to immunotherapy efficacy, there are also CNS-specific barriers that must be considered in this patient population. Applying epigenetic-targeted therapies to enhance the efficacy of immunotherapies could increase the prevalence and severity of immune-related adverse events, if combining these agents work in pediatric brain tumors as they have worked in preclinical models and in non-CNS clinical trial cohorts. Because of the physical space constraints within the CNS, augmenting therapy-related immune responses (i.e. edema and swelling) may damage healthy nervous system tissue or lead to herniation and must be done with caution (201, 202).

#### Neurological immune-related adverse events

While N-irAEs are serious and can be fatal, they are rare; with high-grade N-irAEs having an incidence below 1% (203). Of the high-grade N-irAEs that do occur, common events include myositis, myasthenia, encephalopathies, severe headaches, meningitis, and Guillain-Barre syndrome. In most cases, these patients can be safely managed using therapy interruption and corticosteroids, and many patients can be rechallenged with immunotherapies upon neurologic recovery (203–205). Due to the heightened risk of using immunotherapy in patients with CNS malignancies, caution should be exercised when using epigenetic therapies to sensitize to immunotherapies. Of the clinical trials reviewed here, however, a common theme emerged, where participants who did not develop pseudoprogression (or radiological evidence of an immune response) often also failed to respond to therapy (77, 82, 110). Thus, future advancements that can help predict immunotherapy responsiveness may aid in identifying patients who would benefit from epigenetic pre-treatment, while avoiding the potential increased risk it may pose to natural responders.

#### Immune cell trafficking

Though the CNS was once considered an immune-privileged site, due to the blood-brain barrier, it is now appreciated that the CNS has a dynamic and complex interaction with the immune system (206–208). Notably, the CNS interacts with the peripheral immune system at discrete locations, including in the stroma of the choroid plexus and in the subarachnoid and perivascular spaces (201, 209). Thus, the interaction between the peripheral immune system and the brain parenchyma is tightly regulated. Production of various cytokines and chemokines creates a complex recruitment mechanism for the tumor, affording cancer with the capacity to tailor its immune microenvironment to support its growth. Epigenetic agents that alter the cytokine milieu in a way that modulates immune cell trafficking, infiltration, and activity could be applied to overcome this barrier.

### Role of radiation

Many pediatric brain tumor clinical trials incorporate radiation as a standard-of-care baseline, necessitating careful consideration of how epigenetic therapy, radiation therapy, and immunotherapy can be optimally sequenced. There is evidence that chromatin compaction can impact sensitivity to radiation as well as efficiency of DNA double-strand break repair, and that epigenetic agents that alter chromatin compaction, such as HDAC inhibitors, can influence radiosensitivity (210). The DNA demethylating agent, zebularine, has also been shown to enhance radiosensitivity *in vitro*, including in the glioma cell line U251. Mechanistically, zebularine was shown to prolong repair of radiation-induced DNA damage (211). *In vivo*, zebularine treatment prior to radiation administration prolonged survival. The optimal timing of demethylating therapy relative to radiation administration was not assessed in this study, however (211). Additionally, limited preclinical evidence suggests that radiation may reduce global DNA methylation (212, 213). Thus, it is possible that lead in therapy with epigenetic agents may alter chromatin architecture in a way that increases susceptibility to radiation-induced DNA damage; radiation may also augment tumor immunogenicity induced by epigenetic agents. Additionally, given that perturbations of the epigenome by some inhibitors have been shown to be transient, studies may benefit from prolonged use of low-dose epigenetic therapies (214, 215). Thus, future trials may use relatively higher dose epigenetic therapies as a lead in, before switching to concurrent use of very low dose epigenetic-targeted inhibitors with radiotherapy and immunotherapy. Caution should be exercised when designing and implementing such clinical trials, and therapy should be ceased when dose-limiting toxicities, such as myelosuppression for decitabine, or NirAEs are observed (216). Further studies are necessary to delineate the complex and potentially synergistic interactions of epigenetic therapy, radiation, and immunotherapy to maximize clinical benefit for pediatric brain tumors.

## Conclusion

The prognosis of high-grade pediatric brain tumors continues to be poor, and these tumors are in desperate need of novel approaches targeting multiple aspects of tumor biology. Immunotherapies have been revolutionary for adult cancers, and their application to pediatric brain tumors remains a promising approach. Several features of the immune microenvironment of pediatric brain tumors hinder immunotherapy efficacy, however. Some tumors, like DMG are immunoquiescent and have low lymphocytic infiltration, high infiltrating myeloid cells, and low expression of MHC (42). Other examples, such as ATRT are characterized by higher lymphocyte infiltration, but also have high expression of immune checkpoint molecules, thus dampening immune-mediated clearance of the tumor (26).

Full efficacy of immunotherapy approaches must overcome these hostile aspects of the TME. Indeed, while immunotherapy approaches such as oncolytic viruses, dendritic cell vaccines, CAR T cells, and immune checkpoint blockade are promising in some patients, significant hurdles must be overcome before these therapies can significantly improve disease prognosis. For example, an immunosuppressive environment, low effector cell function, low baseline immunogenicity of pediatric CNS malignancies, and CNS-specific barriers, such as NirAEs have all been identified as barriers to efficacy.

Epigenetic targeted agents can modulate the transcriptional profile of several aspects of the TME and have the potential to revolutionize the application of immunotherapies for pediatric patients with CNS malignancies. The use of agents that modulate histone acetylation, DNA methylation, and histone methylation can reshape immunosuppressive myeloid cell populations, enhance effector function of NK and T cells, increase neoantigen expression on cancer cells, and induce viral mimicry. These approaches have been shown to synergize with immunotherapy in preclinical models, and there is some indication that these therapies also hold promise in clinical trials. Use of dual epigenetic treatment and immunotherapy should be exercised with caution to avoid NirAEs, however, this approach could transform immunotherapy use in the hands of experienced providers.

Practically, there are two classes of epigenetic marks (DNA methylation and histone acetylation) that can be targeted by FDA approved drugs, and these small molecules could be integrated into future clinical trials using immunotherapies. Unfortunately, tazemetostat, an EZH2i and the only FDA approved agent that targets histone methylation, was recently withdrawn from the market by the manufacturer due to an increase in therapy-related secondary malignancies. Given that EZH2 dysregulation is critical to the normal biology of immune cells and the pathogenesis of several pediatric brain tumors, efforts should be undertaken to see if and how these inhibitors could be used safely in the future (41, 172, 217). It is unclear whether secondary malignancies caused by tazemetostat are due to on- or off-target effects of the drug, and additional efforts should be made to better understand the pharmacokinetics and pharmacodynamics of EZH2 inhibitors and their selectivity.

Future clinical trials could use FDA-approved small molecules that target DNA methylation or histone acetylation as monotherapy or combination therapy prior to immunotherapy administration. Decitabine is an FDA approved DNMT inhibitor that has demonstrated brain penetrance in preclinical models (200). Vorinostat and valproic acid are FDA approved HDAC inhibitors that have demonstrated brain penetrance (218, 219). Additional efforts should be taken to move other epigenetic-targeting agents, such as LSD1 inhibitors, BET inhibitors, and G9a inhibitors towards clinical use to expand the available options. Our review of preclinical and clinical work strongly suggests that epigenetic pretreatment will remodel the tumor microenvironment in a way that enhances immunotherapy efficacy.

Finally, we would like to comment on the potential for future immunotherapy trials to focus on shared targets that are redundantly overexpressed following epigenetic targeted therapies. It has been shown that, under therapeutic pressure from immunotherapies that target one/few antigens, clones with loss of the target antigen have a competitive advantage, enabling therapeutic resistance; this phenomenon has been termed immunoediting (220). For example, loss of NY-ESO-1 due to vaccination pressure has been shown in a sarcoma patient (NCT02775292) and in a metastatic melanoma patient to lead to resistance (221, 222). Perhaps, designing immunotherapies that target multiple CTAs would enhance the durability of early responses. Preclinical evidence suggests that this end might be facilitated with the use of epigenetic priming. It has been demonstrated that several CTAs, including members of the MAGE, PAGE, and SSX families are upregulated consistently across multiple tumor types (breast, colorectal, and ovarian) in response to hypomethylating treatment with azacitidine (149).

The reviewed preclinical evidence suggests that pre-treatment with decitabine could upregulate consistent antigens that could be directly targeted or could enhance a whole tumor-lysate derived dendritic cell vaccine. The identification of neoantigen targets that are consistently upregulated by epigenetic therapies across patient samples could conceivably lead to the development of an off-the-shelf product manufactured using redundant neoantigen targets. These vaccines could then be used in patients pre-treated with decitabine, which has been shown to be brain penetrant (200). While this is a compelling hypothesis, further work is warranted to investigate the feasibility and translational potential of such an approach. While consistent neoantigen expression has been reported between tumor types *in vitro*, further work would need to validate this in patient samples (149). We expect that there may be variability in epigenetically-induced upregulation of such targets, as is the case for *NY-ESO-1* and *MAGEA3* expression in clinical AML samples post-decitabine treatment, and that patients would need to be screened for the neoantigen targets (223).Taken together, epigenetic therapy has the potential to enhance the efficacy of immunotherapy, but significant work will be needed to verify the translational potential of such an approach.

Looking forward, review of existing clinical and preclinical evidence indicates that epigenetic-targeted therapies could play a pivotal role in enhancing immunotherapy responses, and this approach warrants dedicated evaluation in pediatric brain tumor patients. Additional efforts should be aimed at improving the safety profile, availability, and brain penetrance of epigenetic targeted agents that are not yet in clinical use. With thoughtful trial design, epigenetic therapies have the potential to meaningfully reshape immunotherapy outcomes for pediatric patients.
